# Collagen IX gene polymorphisms and lumbar disc degeneration: a systematic review and meta-analysis

**DOI:** 10.1186/s13018-018-0750-0

**Published:** 2018-03-05

**Authors:** Huihong Wu, Siting Wang, Weiyou Chen, Xinli Zhan, Zengming Xiao, Hua Jiang, Qingjun Wei

**Affiliations:** 1 Division of Spine Surgery, The First Affiliated Hospital of Guangxi Medical University, No.6 Shuangyong Road, Nanning, 530021 China; 2 Department of Orthopaedic Surgery, The First Affiliated Hospital of Guangxi Medical University, Nanning, China

**Keywords:** COL9A2, COL9A3, Lumbar disc disease, Meta-analysis, Polymorphisms

## Abstract

**Background:**

An increasing number of studies have investigated associations between collagen IX alpha 2 chain (COL9A2) and collagen IX alpha 3 chain (COL9A3) gene polymorphisms and the risk of lumbar disc degeneration (LDD). However, these studies have yielded contradictory results. The purpose of this meta-analysis is to investigate the association between the collagen IX gene polymorphisms (rs12077871, rs12722877, rs7533552 in COL9A2; rs61734651 in COL9A3) and LDD.

**Methods:**

All relevant articles were collected from PubMed, Web of Science, and China National Knowledge Infrastructure (CNKI). The last electronic search was performed on September 1, 2017. The allele/genotype frequencies were extracted from each study. The odds ratio (OR) and 95% confidence interval (CI) were used to assess the strength of associations under the five comparison genetic models. Statistical analysis was performed by Review Manager (RevMan) 5.31 software.

**Results:**

The meta-analysis of 10 case-control studies, including 2102 LDD cases and 2507 controls, indicated that COL9A2 gene (rs12077871, rs12722877, rs7533552) and COL9A3 gene (rs61734651) polymorphisms were not associated with LDD (rs12077871: T vs. C, OR = 1.85, 95% CI = 0.87–3.91, *P* = 0.11; rs12722877: G vs. C, OR = 0.83, 95% CI = 0.69–1.01, *P* = 0.06; rs7533552: G vs. A, OR = 1.11, 95% CI = 0.98–1.25, *P* = 0.09; rs61734651: T vs. C, OR = 1.57, 95% CI = 0.51–4.84, *P* = 0.43). The Egger text and the Begg funnel plot did not show any evidence of publication bias.

**Conclusion:**

rs12077871, rs12722877, and rs7533552 variants in COL9A2 and rs61734651 variant in COL9A3 were not significantly associated with a predisposition to LDD. Large-scale and well-designed studies are needed to confirm this conclusion.

## Background

Low back pain (LBP) is a prevalent disease in adults, especially those ranging from 40 to 80 years of age [1]. LBP can be classified into two types: acute and chronic. Chronic LBP is characterized by persistent pain and a high risk of disability, which contributes to soaring medical costs and loss of labor, possibly leading to a critical impact on the social economy [2]. The major cause of LBP is lumbar disc degeneration (LDD) [3]. Although the pathogenesis of LDD is multivariate, genetic factors have been reported to play an important role in LDD, including collagen I alpha 1 (COL1A1) gene, collagen IX alpha 1 chain (COL9A1) gene, collagen IX alpha 2 chain (COL9A2) gene, collagen IX alpha 3 chain (COL9A3) gene, vitamin D receptor gene, and cartilage intermediate-layer protein gene [4–7].

Collagen IX gene is one of the most recent trending research targets among numerous genes [8]. Collagen IX has been demonstrated to serve as a bridge between collagenous and non-collagenous proteins in normal tissues [9]. COL9A2 and COL9A3 genes encode α2 and α3 chains on collagen IX, respectively [10, 11]. To date, numerous groups have reported the associations of COL9A2 and COL9A3 gene polymorphisms with the risk of LDD. The first study, conducted by Annunen et al., suggested that COL9A2 gene polymorphism (rs12077871) was associated with LDD in the Finnish population [4]. Another study by Paassilta et al. in Finland suggested that rs12077871 was not relevant to LDD, but rs61734651 in COL9A3 gene was relevant to LDD [12]. A number of studies have been conducted on this topic. However, the results were generally inconsistent and inconclusive. Therefore, we collected all the relevant studies, including 2102 cases and 2507 controls, to perform a meta-analysis in order to investigate the association between COL9A2 and COL9A3 gene polymorphisms and LDD predisposition.

## Methods

### Strategy for literature search

The study was conducted by searching literature databases, including PubMed (http://www.ncbi.nlm.nih.gov/pubmed), Web of Science (http://www.isiknowledge.com/), and CNKI (China National Knowledge Infrastructure). To identify all possible studies, we used the following terms: “LDD” or “Lumbar disc disease” or “Lumbar disc degeneration” and “COL9A2” or “COL9A3” or “Collagen IX” or “Polymorphisms” or “COL9A*.” No language or publication date restrictions were applied. The last electronic search was performed on September 1, 2017.

### Inclusion and exclusion criteria

The following inclusion criteria were used to search eligible studies: (1) investigated the relationship of COL9A2 or COL9A3 gene polymorphisms with LDD, (2) case-control or cohort design, and (3) provided available data for the estimation of an odds ratio (OR) and 95% confidence interval (CI). Studies were excluded according to the following criteria: (1) repeated publications, (2) reported in the form of comment and review, (3) irrelevant to LDD, and (4) unavailable allele and genotype frequencies. Two investigators (W.H.H and W.S.T) independently evaluated the articles for compliance with the inclusion and exclusion criteria. For disagreements, a consensus was reached by a third investigator (C.W.Y).

### Data extraction

The following data were extracted from all the eligible articles: first author’s name, publication year, country of enrollment, ethnicity, numbers of cases and controls, disease, diagnostic criteria, source of controls, genotyping methods, genotypes, and allele frequency of cases and controls. Data were extracted independently by two reviewers (W.H.H and W.S.T), and a third reviewer (C.W.Y) was needed for any disagreements.

### Methodological quality

The methodological quality of the included studies was evaluated according to a quality evaluation form base in the Critical Appraisal Skills Programme (CASP) for case-control study [13]. The assessment of CASP contains 10 questions, which are associated with information given by single studies. There are three degrees for each question: “yes” (scored 2), “can’t tell” (scored 1), or “no” (scored 0). The maximum score is 20, and the minimum score is 0. Studies could be divided into three grades: grade A (high quality, scored 15–20), grade B (medium quality, scored 8–14), and grade C (low quality, scored 0–7).

### Statistical analysis

Meta-analysis was conducted using Revman 5.31 software (Nordic Cochrane Centre, Cochrane Collaboration, Copenhagen, Denmark). The pooled OR and 95% CI were used to estimate the strength of correlations between COLA9A2 (rs12077871, rs12722877, rs7533552) and COL9A3 (rs61734651) variants and LDD. Heterogeneity was tested using the chi-square-based *Q* test and *I*^2^ test. To calculate the pooled OR, a fix effect model was performed if no heterogeneity existed (*P* > 0.05, *I*^2^ < 50%). Otherwise, a random effect model was used. Five comparison genetic models were conducted to evaluate the association between the four single nucleotide polymorphisms (SNPs) (rs12077871, rs12722877, rs7533552, rs61734651) and LDD risk. Hardy-Weinberg equilibrium among controls was estimated using the HWE version 1.20 program (Columbia University, New York, NY). If there was heterogeneity in some models (*P* < 0.05, *I*^2^ > 50%), we performed the sensitivity test to assess the possible influence of one study on the pooled OR. Studies were removed, in turn, from the overall analysis. In addition, we performed subgroup analysis stratified by ethnicity. Funnel plots and Egger’s tests were used to assess the potential publication bias.

It will be reasonable to estimate the combined effect from a group study if the effects found individually in studies are sufficiently similar. Some variation between the studies is expected because the estimates of the treatment effect are influenced by chance. What we need to know is whether there is more than just a chance-related variation. The heterogeneity test was performed to determine this extreme variation. Therefore, in this study, chi-square statistic was performed together with the degree of freedom. During the meta-analysis, the results were evaluated by incorporating suspicious relevance studies into statistical analyses. Then, these studies, the appropriateness of which were questionable, were excluded from the study and the same analyses were repeated. After comparing the two results, the data for the appropriate ones were interpreted.

## Results

### Characteristics of studies

**Fig. 1:**
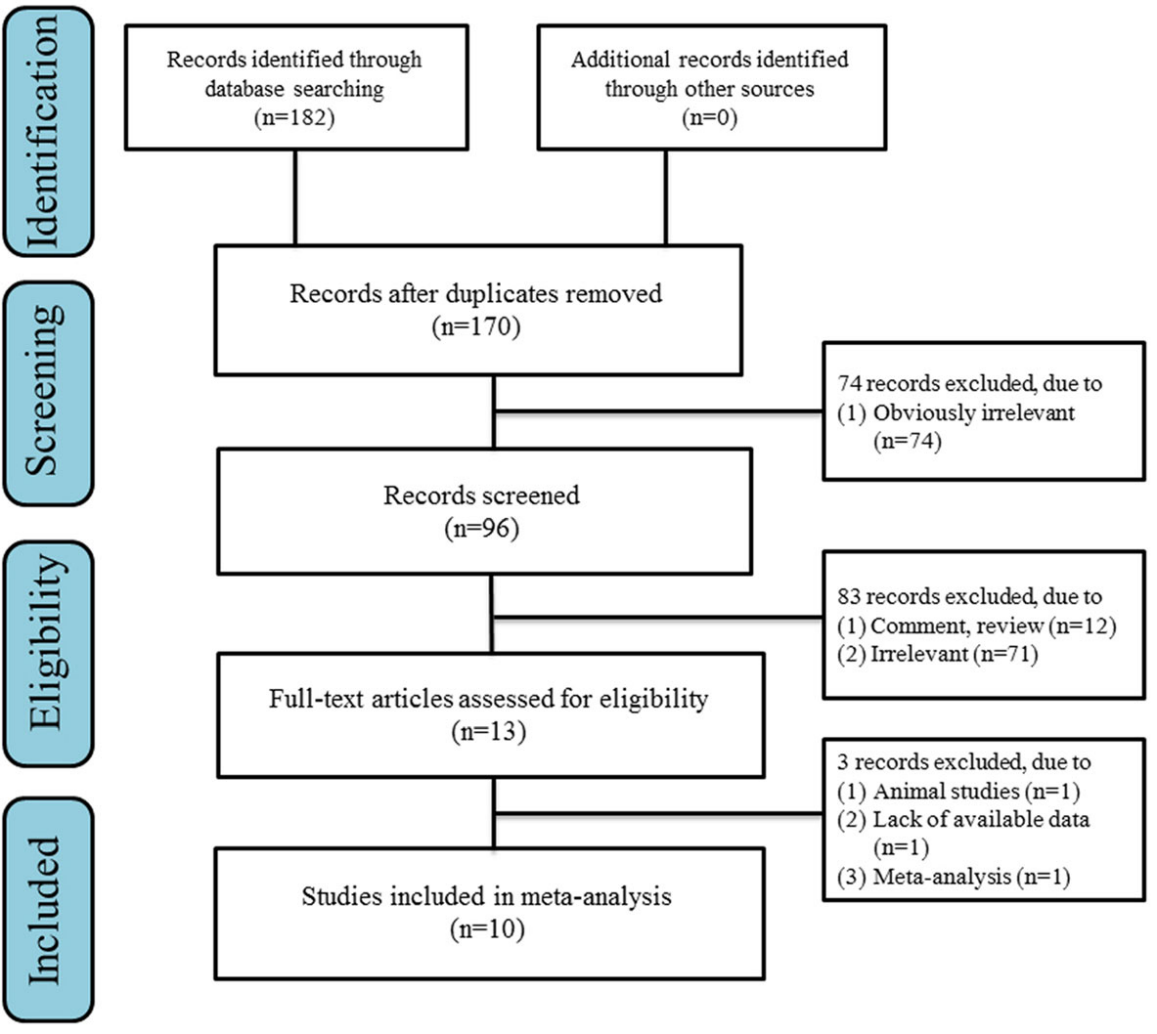
Literature search flow diagram

As shown in Fig. 1, 182 potentially relevant studies were searched from the electronic database. Ten studies were identified by screening the full article, which included 2102 cases and 2507 controls (rs12077871 6 studies, 1086 cases, and 1210 controls; rs12722877 4 studies, 1030 cases, and 1235 controls; rs7533552 5 studies, 1172 cases, and 1287 controls; rs61734651 4 studies, 365 cases, and 631 controls). Of those, seven articles reported that gene polymorphism in two or more loci were associated with LDD risk. Table 1 and Table 2 show the main characteristics of included studies. The results of quality assessment are also shown in Table 1. All included studies were categorized as grade A, with scores ranging from 18 to 20.

**Table 1:** Characteristics of the case-control studies included in systematic review

First author	Year	Population	Ethnicity	Number of cases/controls	Disease	Diagnostic criteria	Control group	Genotyping determination	CASP
rs12077871									
Annunen et al. [4]	1999	Finland	Caucasian	157/174	LDD	MRI, CT	Healthy normal without spine-related problems	PCR-seq	18
Noponen-Hietala et al. [15]	2003	Finland	Caucasian	29/56	LDD	MRI, CT	Patients without spine-related problems	PCR-seq CSGE	20
Seki et al. [17]	2006	Japan	Asian	470/654	LDD	MRI, PR	NM	PCR-seq	19
Song et al. [18]	2010	China	Asian	125/125	LDD	MRI, PR	Patients without LDD	TaqMan assay	18
Hyun et al. [14]	2011	Korean	Asian	205/101	LDD	MRI	Patients without spine-related problems	PCR-seq	20
Rathod et al. [16]	2012	India	Asian	100/100	LDD	MRI	Patients without spine-related problems	TaqMan assay	19
rs12722877									
Paassilta et al. [12]	2001	Finland	Caucasian	156/167	LDD	MRI, CT	Healthy normal, patients with osteoarthritis	PCR-seq CSGE	20
Seki et al. [17]	2006	Japan	Asian	469/654	LDD	MRI, PR	NM	PCR-seq	19
Song et al. [18]	2010	China	Asian	125/126	LDD	MRI, PR	Patients without LDD	TaqMan assay	18
Chen et al. [19]	2013	China	Asian	280/268	LDD	MRI, CT, PR	Patients without LDD	TaqMan assay	18
rs7533552									
Annunen et al. [4]	1999	Finland	Caucasian	157/174	LDD	MRI, CT	Healthy normal, patients with osteoarthritis	PCR-seq	18
Seki et al. [17]	2006	Japan	Asian	470/654	LDD	MRI, PR	NM	TaqMan assay	19
Song et al. [18]	2010	China	Asian	125/125	LDD	MRI, PR	Patients without LDD	TaqMan assay	18
Hyun et al. [14]	2011	Korean	Asian	205/101	LDD	MRI	Patients without spine-related and arthritic problems	PCR-seq	20
Meng et al. [20]	2016	China	Asian	215/230	LDD	MRI, CT	Healthy	PCR-seq	19
rs61734651									
Paassilta et al. [12]	2001	Finland	Caucasian	156/167	LDD	MRI, CT	Healthy normal, patients with osteoarthritis	PCR-seq CSGE	20
Noponen-Hietala et al. [15]	2003	Finland	Caucasian	29/56	LDD	MRI, CT	Patients without spine-related problems	PCR-seq CSGE	20
Eskola et al. [21]	2010	Danish	Caucasian	154/66	LDD	MRI	Patients without spine-related problems	PCR-seq	19
Rathod et al. [16]	2012	India	Asian	100/100	LDD	MRI	Patients without spine-related problems	TaqMan assay	19

*NM* not mentioned, *MRI* magnetic resonance imaging, *CT* computerized tomography, *PR* plain radiographs, *CASP* Critical Appraisal Skills Programme

**Table 2:** Genotype and allele frequency of COL9A2 and COL9A3 gene polymorphisms in LDD patients and controls

Study			Case group					Control group					HWE for control
Author	Year	Ethnicity	11	12	22	1	2	11	12	22	1	2	
rs12077871 (C vs. T)													
Annunen et al. [4]	1999	Caucasian	NM	NM	NM	308	6	NM	NM	NM	348	0	
Noponen-Hietala et al. [15]	2003	Caucasian	28	1	0	57	1	56	0	0	112	0	1.000000
Seki et al. [17]	2006	Asian	370	91	9	831	109	504	136	14	1144	164	0.184822
Song et al. [18]	2010	Asian	95	30	0	220	30	100	24	1	224	26	0.735473
Hyun et al. [14]	2011	Asian	155	46	4	356	54	76	22	3	174	28	0.377303
Rathod et al. [16]	2012	Asian	43	43	15	128	72	83	17	0	183	17	0.352901
rs12722877 (C vs. G)													
Paassilta et al. [12]	2001	Caucasian	NM	NM	NM	290	22	NM	NM	NM	312	22	
Seki et al. [17]	2006	Asian	414	54	1	882	56	576	76	2	1228	80	0.761095
Song et al. [18]	2010	Asian	103	22	0	228	22	109	17	0	235	17	0.416784
Chen et al. [19]	2013	Asian	158	98	24	414	146	120	113	35	353	183	0.306985
rs7533552 (C vs. G)													
Annunen et al. [4]	1999	Caucasian	NM	NM	NM	228	86	NM	NM	NM	258	90	
Seki et al. [17]	2006	Asian	217	221	32	655	285	327	277	50	931	377	0.40912
Song et al. [18]	2010	Asian	21	63	41	105	145	25	67	33	117	133	0.39295
Hyun et al. [14]	2011	Asian	86	97	22	269	141	41	47	13	129	73	0.934487
Meng et al. [20]	2016	Asian	68	113	34	249	181	81	131	18	293	167	0.000449
rs61734651 (C vs. T)													
Paassilta et al. [12]	2001	Caucasian	131	38	2	300	42	291	30	0	612	30	0.3798
Noponen-Hietala et al. [15]	2003	Caucasian	25	3	1	53	5	56	0	0	112	0	1.000000
Eskola et al. [21]	2010	Caucasian	57	9	0	123	9	123	30	1	276	32	0.566482
Rathod et al. [16]	2012	Asian	95	5	0	195	5	93	7	0	193	7	0.716846

11, 12, and 22 indicate CC, TC, and TT for rs12077871; CC, CG, and GG for rs12722877; AA, GA, and GG for rs7533552; CC, TC, and TT for rs61734651, respectively

*NM* not mentioned

### Quantitative data analysis

#### Association of rs12077871 and LDD susceptibility

**Fig. 2:**
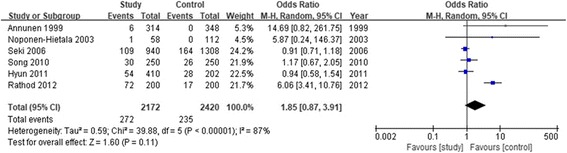
Forest plot of the pooled ORs with 95% CIs for associations between COL9A2 rs12077871 and LDD in overall populations under the allelic contrast model (C vs. T allele); events: the number of C allele

The association between rs12077871 polymorphism and LDD predisposition was determined in six case-control studies [4, 14–18], including 1086 cases and 1210 controls. As shown in Table 3, we evaluated the association between rs12077871 polymorphism and LDD predisposition under five genetic models (T vs. C: OR = 1.85, 95% CI = 0.87–3.91, *P* = 0.11). Furthermore, we performed the subgroup analysis stratified by ethnicity. The result showed rs12077871 was not associated with LDD risk in the Asian population. The subgroup analysis of the Caucasian population was unavailable as there were insufficient studies. The forest plot of the allele contrast genetic model demonstrated the association between rs12077871 polymorphism and LDD susceptibility (Fig. 2).

**Table 3:** Association test and heterogeneity test of COL9A2 and COL9A3 gene polymorphisms (rs12077871, rs12727871, rs7533553, and rs61734657)

SNP	Genetic model	Analysis model	Test of association			Heterogeneity test	
			OR	95% Cl	*P* value	*I*^2^ (%)	*P*_het_
rs12077871							
	Allelic						
	C vs. T	Random	1.85	[0.87,3.91]	0.110	87	< 0.00001
	Codominant model						
	CC vs. TT	Random	1.55	[0.26,9.16]	0.630	74	0.01
	CT vs. TT	Random	1.57	[0.82,3.01]	0.170	81	0.0004
	Dominant model						
	TT + CT vs. CC	Fixed	0.27	[0.06.1.29]	0.010	0	0.5
	Recessive model						
	CC + CT vs. TT	Random	1.7	[0.79,3.64]	0.180	87	< 0.00001
rs12722877							
	Allelic						
	C vs. G	Random	0.90	[0.673,1.213]	0.499	47.70	0.125
	Codominant model						
	CC vs. GG	Fixed	0.53	[0.304,0.923]	0.025	0	0.818
	CG vs. GG	Fixed	0.79	[0445,1.388]	0.406	0	0.927
	Dominant model						
	GG + CG vs. CC	Random	1.13	[0.746,1.722]	0.556	63.70	0.064
	Recessive model						
	CC + CG vs. GG	Fixed	0.63	[0.367,1.072]	0.088	0	0.93
rs7533552							
	Allelic						
	A vs. G	Fixed	1.11	[0.983,1.253]	0.092	0	0.658
	Codominant model						
	AA vs. GG	Random	1.26	[0.807,1.902]	0.310	46.60	0.132
	AG vs. GG	Random	1.17	[0.735,1.867]	0.505	58.60	0.065
	Dominant model						
	GG + AG vs. AA	Fixed	0.88	[0.733,1.050]	0.154	0	0.875
	Recessive model						
	AA + AG vs. GG	Random	1.21	[0.784,1.879]	0.384	56.70	0.074
rs61734651							
	Allelic						
	C vs. T	Random	1.57	[0.51,4.84]	0.430	81	0.001
	Codominant model						
	CC vs. TT	Fixed	3.93	[0.83,18.60]	0.080	0	0.44
	CT vs. TT	Random	1.48	[0.50,4.33]	0.480	77	0.004
	Dominant model						
	TT + CT vs. CC	Fixed	0.27	[0.06,1.29]	0.100	0	0.5
	Recessive model						
	CC + CT vs. TT	Random	1.55	[0.50,4.86]	0.450	80	0.002

*SNP* single nucleotide polymorphism, *OR* odds ratio, *CI* confidence interval; *P*_*het*_ *P* value for heterogeneity, *P < 0.05* statistical significance

#### Association of rs12722877 and LDD susceptibility

**Fig. 3:**
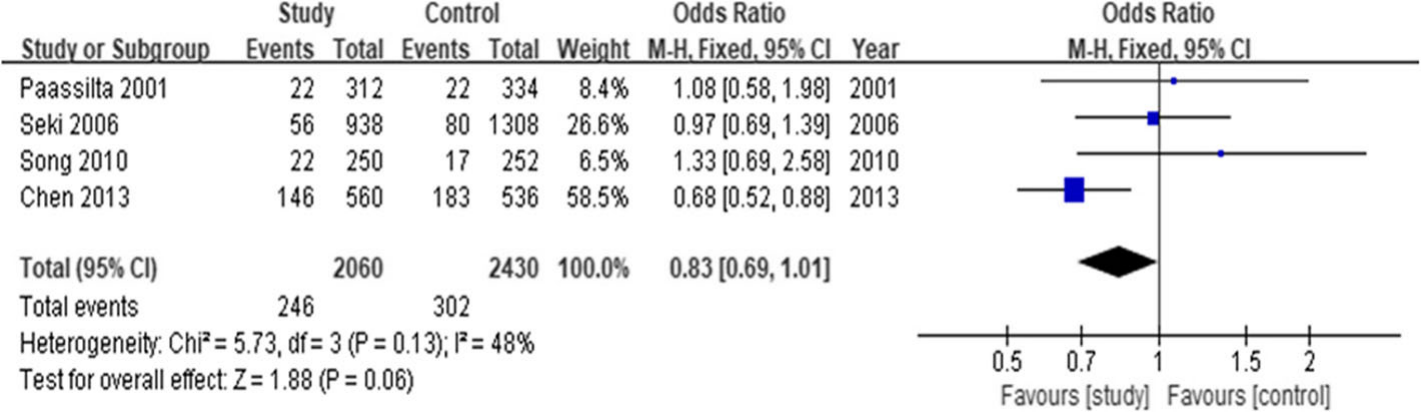
Forest plot of the pooled ORs with 95% CIs for associations between COL9A2 rs12722877 and LDD in overall populations under the allelic contrast model (C vs. G allele); events: the number of C allele

The association between rs12722877 polymorphism and LDD predisposition was determined in four case-control studies [12, 17–19], including 1030 cases and 1215 controls. As shown in Table 3, we used five genetic models to access the relationship between rs12722877 polymorphism and LDD predisposition (G vs. C: OR = 0.83, 95% CI = 0.69–1.01, *P* = 0.06). Moreover, we performed the subgroup analysis stratified by ethnicity. For the Asian population, rs12722877 polymorphism was associated with LDD predisposition under the allele contrast genetic model (OR = 0.81, 95% CI = 0.67–0.99, *P* = 0.04). However, a significant association was not found in other genetic models. The subgroup analysis of the Caucasian population was unavailable due to insufficient studies. The forest plot of the allele contrast genetic model indicated the association between rs12722877 polymorphism and LDD predisposition (Fig. 3).

#### Association of rs7533552 and LDD susceptibility

**Fig. 4:**
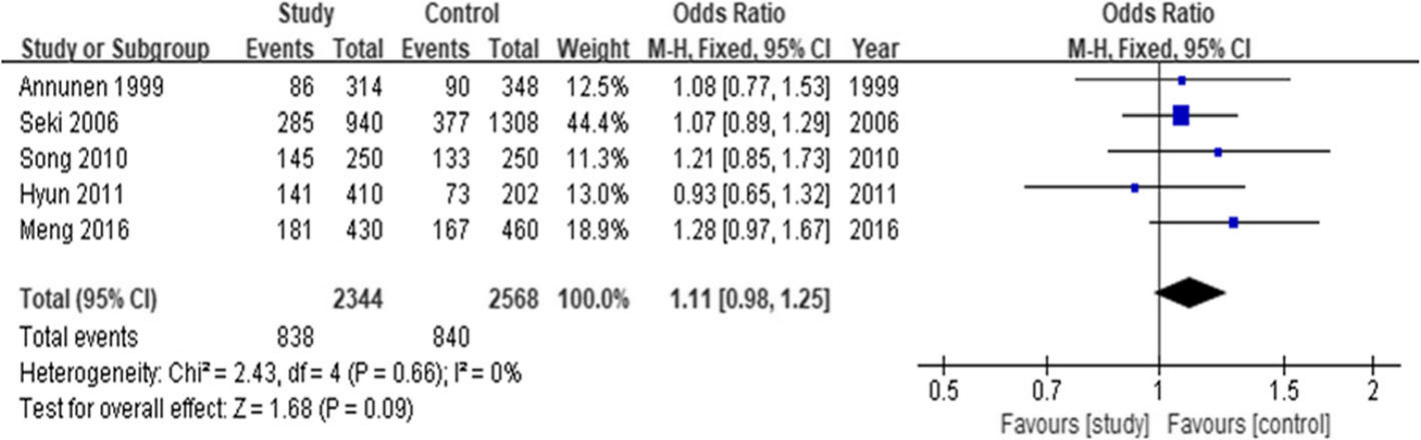
Forest plot of the pooled ORs with 95% CIs for associations between COL9A2 rs7533552 and LDD in overall populations under the allelic contrast model (A vs. G allele); events: the number of A allele

The association between rs7533552 polymorphism and LDD predisposition was determined in five case-control studies [4, 14, 17, 18, 20], including 957 cases and 1054 controls. As shown in Table 3, we implemented five genetic models to access the association between rs7533552 polymorphism and LDD predisposition (G vs. A: OR = 1.11, 95% CI = 0.98–1.25, *P* = 0.09). In addition, we performed the subgroup analysis stratified by ethnicity. The data showed rs7533552 polymorphism was not associated with LDD risk in the Asian population. The forest plot of the allele contrast genetic model demonstrated the association between rs7533552 polymorphism and LDD predisposition (Fig. 4).

#### Association of rs61734651 and LDD susceptibility

**Fig. 5:**
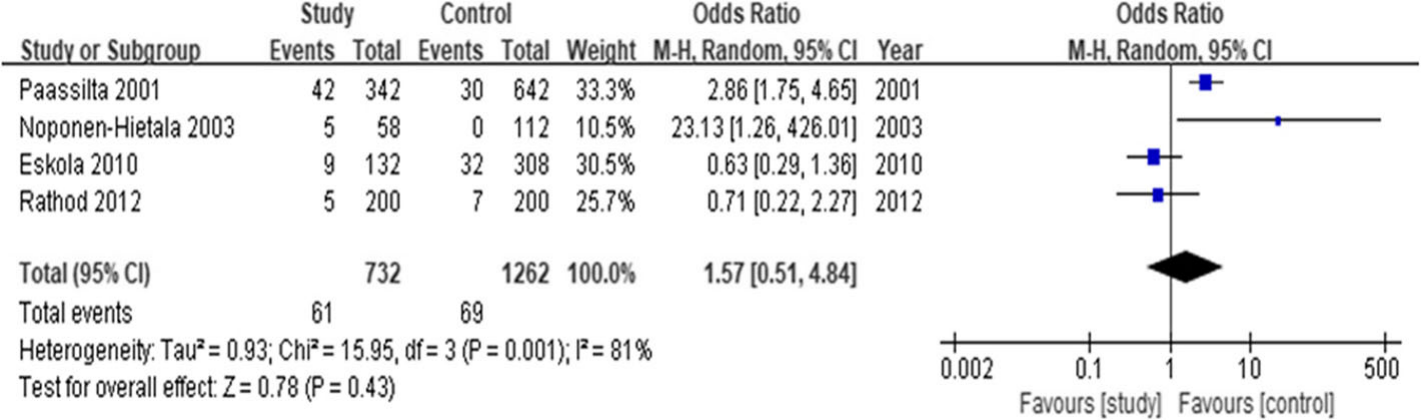
Forest plot of the pooled ORs with 95% CIs for associations between COL9A3 rs61734651 and LDD in overall populations under the allelic contrast model (T vs. C allele); events: the number of C allele

The association between rs61734651 polymorphism and LDD predisposition was determined in four case-control studies [12, 15, 16, 21], including 365 cases and 631 controls. As shown in Table 3, we evaluated the association between rs61734651 polymorphism and LDD predisposition under five genetic models (T vs. C: OR = 1.57, 95% CI = 0.51–4.84, *P* = 0.43). Furthermore, we performed the subgroup analysis stratified by ethnicity. The result showed rs61734651 polymorphism was not associated with LDD susceptibility in the Caucasian population. The subgroup analysis of the Asian population was unavailable due to insufficient studies. The forest plot of the allele contrast genetic model indicated the association between rs61734651 polymorphism and LDD predisposition (Fig. 5).

### Sensitivity analysis and publication bias

Sensitivity analysis was performed by excluding one study at a time. For the four SNPs, the results did not alter under all genetic models after sensitivity analysis (Table 4). For rs12077871, when we omitted the study reported by Rathod et al. [16], the heterogeneity was obviously reduced under allele contrast genetic models. For rs61734651, when we excluded the study reported by Paassilta et al. [12], the heterogeneity was significantly reduced under the allele contrast genetic model (Table 5). Sensitivity analysis indicated that our results were robust and consistent.

**Table 4:** Heterogeneity analysis and Egger regression analysis of COL9A2 and COL9A3 gene polymorphisms (rs12077871, rs12727871, rs7533553, and rs61734657)

SNP	Analysis model	Heterogeneity analysis			Egger regression analysis		
		*χ* ^2^	*P*	*I*^2^ (%)	*t*	95% CI	*P*
rs12077871	Random	39.88	< 0.0001	87.50	− 1.33	[− 7.57, 2.68]	0.26
rs12722877	Fixed	5.73	0.125	47.70	− 2.78	[− 7.51, 1.61]	0.11
rs7533552	Fixed	1.15	0.776	0.00	0.06	[− 5.26, 5.41]	0.96
rs61734651	Random	15.12	0.002	81.00	0.1	[− 12.0, 12.6]	0.93

**Table 5:** The result of sensitivity analysis with each study omitted for rs12077871 in COL9A2 and rs61734651 in COL9A3

Study omitted	OR	95% CI	*P*
rs12077871 C/T			
Annunen et al. [4]	0.61	[0.29, 1.30]	0.2
Noponen-Hietala et al. [15]	0.57	[0.26, 1.24]	0.15
Seki et al. [17]	0.41	[0.15, 1.14]	0.09
Song et al. [18]	0.45	[0.17, 1.20]	0.11
Hyun et al. [14]	0.42	[0.15, 1.15]	0.09
Rathod et al. [16]	1.00	[0.81, 1.23]	0.99
rs61734651 C/T			
Noponen-Hietala et al. [15]	0.88	[0.30, 2.63]	0.002
Paassilta et al. [12]	0.88	[0.23, 3.33]	0.05
Rathod et al. [16]	0.48	[0.12, 1.93]	0.002
Eskola et al. [21]	0.44	[0.12, 1.65]	0.03

*OR* odds ratio, *CI* confidence interval

**Fig. 6:**
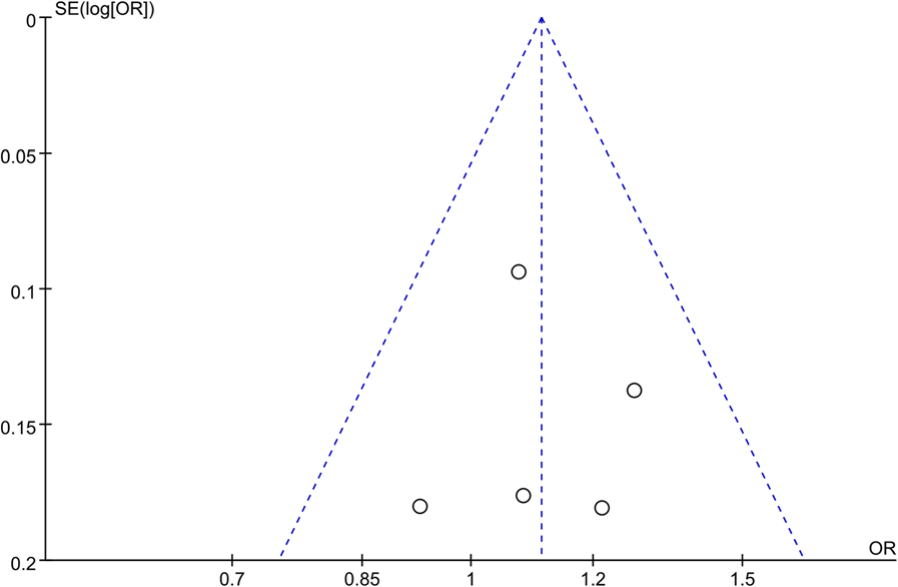
Funnel plot analysis for publication bias; COL9A2 gene polymorphism (rs7533552) under the allelic contrast model

Publication bias was appraised by applying Begg’s funnel plots and Egger’s regression test (Fig. 6). The result indicated no significant publication bias under all genetic models (all *P* > 0.05 for all models tested).

## Discussion

Various risk factors were widely considered to be related to symptomatic LDD, including environmental, ergonomic, and biometric factors. Nowadays, increasing evidence indicates that genetic factors play critical roles in LDD [22]. Plenty of recent studies show associations of COL9A2 gene (rs12077871, rs12722877, rs7533552) and COL9A3 gene (rs61734651) polymorphisms with the incidence of LDD. The study reported by Annunen et al. initially suggested that COL9A2 gene polymorphism was associated with LDD in the Finnish population [4]. Some studies were undertaken to replicate this finding [9, 14, 16]. However, the other studies did not come to the same conclusion [23]. Under the circumstances, a meta-analysis conducted by Zhang et al. investigated the association between COL9A2 gene polymorphism and LDD risk in 2014 [24]. The results indicated no significant association between COL9A2 gene polymorphisms and LDD predisposition. A number of studies have also examined the association between COL9A2 gene polymorphism and LDD in recent years [19, 20]. However, the previous meta-analysis [24] did not include data from the recent studies, which may lead to inaccuracy in the conclusion. The Cochrane Back Review Group has advocated that a comprehensive meta-analysis needs to be updated with data from the latest studies to be timely [25]. Furthermore, COL9A2 and COL9A3 genes respectively encode α2 and α3 chains of collagen IX, indicating a close relationship between COL9A2 and COL9A3. Several studies have reported that COL9A3 gene polymorphism was associated with susceptibility to LDD [10, 26–28]. However, these published studies have yielded contradictory results rather than conclusive evidence [15, 17, 23]. Thus, we performed a meta-analysis on the associations between COL9A2 and COL9A3 gene polymorphisms and LDD susceptibility. To the best of our knowledge, the current study is the largest sample size of meta-analysis to investigate the association between COL9A2 and COL9A3 gene polymorphisms and LDD predisposition.

Several authors reported that collagen IX provided optimal stability to the lumbar disc cross-linked with collagen II [27] and indicated that collagen IX is crucial for the functional lifespan of intervertebral discs [29]. A role for collagen IX in disc degeneration is supported by human and animal studies [2, 12]. Furthermore, the COL9A2 and COL9A3 genes are highly expressed in intervertebral discs and encode the α2 and α3 chains of type IX collagen, which suggests that they are critical for intervertebral disc metabolism [30]. Mutations of COL9A2 and COL9A3 genes could interfere with the bond between collagen IX and collagen II, leading to decreased stability of the lumbar disc [31]. Thus, it remains plausible that COL9A2 and COL9A3 may be involved in the etiology of LDD through the intervertebral disc metabolism. COL9A2 and COL9A3 gene polymorphisms are supposed to have an impact on gene regulation. However, the precise role of these SNPs is still unknown. Functional analysis of the COL9A2 and COL9A3 genes might help elucidate the real genetic effect on the etiopathogenesis of LDD.

Our meta-analysis of 10 studies, involving 2102 LDD cases and 2507 controls, found no statistically significant association between COL9A2 gene (rs12077871, rs12722877, rs7533552) and COL9A3 gene (rs61734651) polymorphisms and LDD risk. The previous studies had reported that COL9A2 and COL9A3 gene polymorphisms were related to LDD predisposition in different ethnic population groups. Therefore, we performed a subgroup analysis stratified by ethnicity (Caucasian and Asian). Our results showed rs12722877 was associated with LDD risk in the Asian population under the allele contrast genetic model (C vs. G), but not under other genetic models. This finding was in partial accordance with a previous meta-analysis study [23]. We should note that heterogeneity existed in our study in interpreting the results of our meta-analysis. For rs12077871 and rs61734651 polymorphisms, significant heterogeneity was found in all genetic models except the dominant model; for rs12722877 polymorphism, heterogeneity was detected in dominant model models, while for rs7533552 polymorphism, heterogeneity was detected in codominant and recessive models. For rs12077871 polymorphism, the heterogeneity detected in the four genetic models was effectively decreased in sensitivity analysis after excluding the study by Rathod et al. For rs61734651 polymorphism, sensitivity analysis suggested that the study of Paassilta et al. was the major source of the heterogeneity. The removal of these datasets did not change the overall results of any genetic models. There are some potential explanations for the presence of heterogeneity, including genetic background, study design, and environment factors. Furthermore, heterogeneity may result from the different phenotype selection and diagnostic criteria of LDD [32]. In view of the heterogeneity, the results of the meta-analysis should be interpreted with caution. A more powerful conclusion needs to be supported by future studies with larger sample sizes.

In our systematic review, increasing the number of studies, which were examined, was possible by extending our search criteria. However, we think that this situation may give rise to further confusion among the results and it may prevent making binding inferences. Several limitations of this study should be acknowledged. First, only English and Chinese documents were searched, while reports in other languages were excluded. This may lead to publication bias. Second, the limited sample size of the pooled studies may exert an influence on their statistical power. Third, we did not perform stratification analysis by age, gender, and environmental factors as a data limitation.

## Conclusions

COL9A2 gene (rs12077871, rs12722877, rs7533552) and COL9A3 gene (rs61734651) polymorphisms were not associated with susceptibility to LDD. The associations of COL9A2 and COL9A3 gene polymorphisms and the risk of LDD could not be fully excluded. Large-scale and well-designed studies are needed to further analyze this field.

## Abbreviations

COL9A2: Collagen IX alpha 2 chain
COL9A3: Collagen IX alpha 3 chain
LBP: Low back pain
LDD: Lumbar disc degeneration
SNP: Single nucleotide polymorphism
